# Three-decade trends in nonalcoholic fatty liver disease across 204 countries and territories: An age-period-cohort analysis from 1990 to 2021

**DOI:** 10.1097/MD.0000000000050989

**Published:** 2026-10-02

**Authors:** Meiying Zheng, Meng Zou, Haiying Chen, Luyao Gong, Na Wang, Zhaojie Su

**Affiliations:** a Department of Infection, the 910th Hospital of the PLA Joint Logistics Support Force, Quanzhou, Fujian, China; b Department of General Surgery, Songgang People’s Hospital, Shenzhen, Guangdong, China; c Department of Nursing, the 910th Hospital of the PLA Joint Logistics Support Force, Quanzhou, Fujian, China; d Department of Pancreatic, Hepatobiliary and Gallbladder Surgery, The Sixth Affiliated Hospital of Sun Yat-sen University, Guangzhou, Guangdong, China; e Department of General Surgery, Xiang’an Hospital of Xiamen University, Xiamen, Fujian, China.

**Keywords:** age-period-cohort, global burden of disease study, global health, incidence, nonalcoholic fatty liver disease

## Abstract

Nonalcoholic fatty liver disease (NAFLD) has emerged as a leading chronic liver disease globally, driven by the rising incidence of obesity, type 2 diabetes, and metabolic syndrome. Despite the increasing burden, most studies focus on prevalence rather than incidence, leaving a gap in understanding temporal patterns of NAFLD incidence. We conducted an age-period-cohort analysis of NAFLD incidence across 204 countries and territories from 1990 to 2021, utilizing data from the Global Burden of Disease Study 2021. Incidence rates were standardized per 100,000 person-years and analyzed by age, sex, region, and socio-demographic index (SDI). The global incidence of NAFLD nearly doubled from 24.9 million cases in 1990 to 48.4 million in 2021. The age-standardized incidence rate in 2021 was 593.28 per 100,000 population (95% confidence interval: 542.72–643.7). Middle SDI regions recorded the highest incidence rate (695/100,000), while low SDI regions had the lowest (483/100,000). The age-period-cohort model indicated a net drift of 0.56% (95% confidence interval: 0.53–0.6) globally. NAFLD incidence has risen substantially over the past 3 decades, particularly in middle-income regions. These findings highlight the need for targeted public health interventions and further research to address the growing global burden of NAFLD.

## 1. Introduction

Nonalcoholic fatty liver disease (NAFLD) has become a leading chronic liver condition globally, largely driven by rising rates of obesity, type 2 diabetes, and metabolic syndrome.^[1]^ As these diseases have rapidly increased in recent decades, NAFLD prevalence has surged, presenting significant public health challenges.^[2]^ NAFLD not only affects liver health but also carries risks for progression to more severe conditions such as nonalcoholic steatohepatitis, cirrhosis, and hepatocellular carcinoma.^[3]^

While NAFLD prevalence has been extensively studied, incidence data remain scarce. Prevalence studies inform the disease burden but do not capture the rate of new cases. Incidence data are crucial for understanding disease patterns, identifying emerging trends, and evaluating the effectiveness of public health measures. These data also reflect shifts in population-level risk factors, such as diet and physical activity, which are linked to NAFLD.^[4,5]^

Recent evidence highlights the role of public health strategies in mitigating NAFLD progression and incidence, including health education, dietary counseling, and lifestyle interventions.^[6]^ Nurse-led initiatives targeting patient education and lifestyle changes have shown promise in reducing risk and slowing progression.^[7,8]^ These efforts, when combined with community-based health programs, can significantly improve population health outcomes by addressing underlying risk factors. However, much of the research on NAFLD incidence is region-specific, leading to fragmented global data.^[2]^ The lack of comprehensive long-term incidence data complicates understanding how economic and lifestyle shifts have influenced NAFLD incidence.^[9]^

This research seeks to address this gap by examining the trends in NAFLD incidence from 1990 to 2021 across 204 countries and territories through the application of an age-period-cohort (APC) model. The APC model efficiently distinguishes the influences of age, period, and cohort, offering valuable insights into the underlying factors contributing to incidence trends.^[10,11]^ This analysis will offer a comprehensive view of NAFLD trends over the past 3 decades, informing global public health strategies to combat this growing disease burden.

## 2. Materials and methods

### 2.1. Data source

This study utilized data from the Global Burden of Disease (GBD) 2021 database, which integrates information from over 100,000 sources, including academic publications, disease registries, household surveys, and clinical data, ensuring a comprehensive representation of global epidemiology.^[12]^ Specifically, the GBD 2021 database provides modeled estimates of NAFLD incidence across age groups, sexes, and geographical locations using standardized estimation procedures. Disease burden estimates for NAFLD were generated using the disease modeling meta-regression version 2.1 and spatio-temporal Gaussian process regression models, which account for variations across age groups, sex, and geographical regions through a Bayesian framework. Additionally, the socio-demographic index (SDI), a composite measure of income, education, and fertility rates, was used to stratify regions and assess trends across different levels of socioeconomic development.^[13]^

### 2.2. Data analysis

#### 2.2.1. Analysis of trends in NAFLD incidence

Temporal trends in NAFLD incidence were assessed by considering both all-age incidence rates (AAIR) and age-standardized incidence rates (ASIR) across all age groups, with a particular focus on age-specific trends. The incidence rates were age-standardized per 100,000 person-years based on the global population structure employed in GBD 2021. Additionally, the incidence distribution was explored across 9 age categories (e.g., 15–19, 20–24, 25–29, …, 50–69, 70+ years) to identify the proportion of cases in each age group and observe any notable variations.

### 2.3. APC modeling

To assess the temporal and generational influences on NAFLD incidence, we employed the APC model. This statistical approach allows for the separation of age, period, and cohort effects, offering a deeper understanding of the dynamics driving the disease incidence over time.^[10]^ The APC model enables the identification of net drift, representing the overall annual change in disease rates, and local drift, which reflects age-specific variations in incidence. By analyzing relative risks, the model quantifies the impact of temporal and cohort influences on NAFLD incidence, where values >1 indicate an increased risk and those <1 suggest a decreased risk.

To mitigate the issue of collinearity between the APC variables, the intrinsic estimator method was used, which ensures the independent estimation of these effects despite their linear dependency.^[11]^ The APC analysis incorporated data on NAFLD incidence from the GBD 2021, which spanned 16 age groups (15–19 to 90–94 years) and 21 overlapping birth cohorts from 1896 to 1904 to 1996 to 2004. The model was applied through the web tool provided by the National Cancer Institute,^[14,15]^ with visualizations created through R statistical software (version 4.4.2; R Foundation for Statistical Computing). Statistical significance was determined using 2-tailed tests, with a *P* value threshold of .05.

## 3. Results

### 3.1. Trends in NAFLD incidencer, 1990 to 2021

The incidence data presented in Figures 1A, B and 2, Table 1, and Table S1, Supplemental Digital Content 1 includes population figures, total incidence counts, AAIR, ASIR, and the net drift of incidence rates over the study period. Between 1990 and 2021, the global incidence of NAFLD rose from 24,856,159 cases (95% confidence interval [CI]: 22,579,697.49–27,333,109.87) to 48,353,272 cases (95% CI: 44,229,138.51–52,358,017.44). In 2021, the ASIR was 593.28 per 100,000 individuals (95% CI: 542.72–643.7). During this period, a net drift of 0.56 (95% CI: 0.53–0.6) in NAFLD incidence was estimated by the APC model.

**Figure 1.**
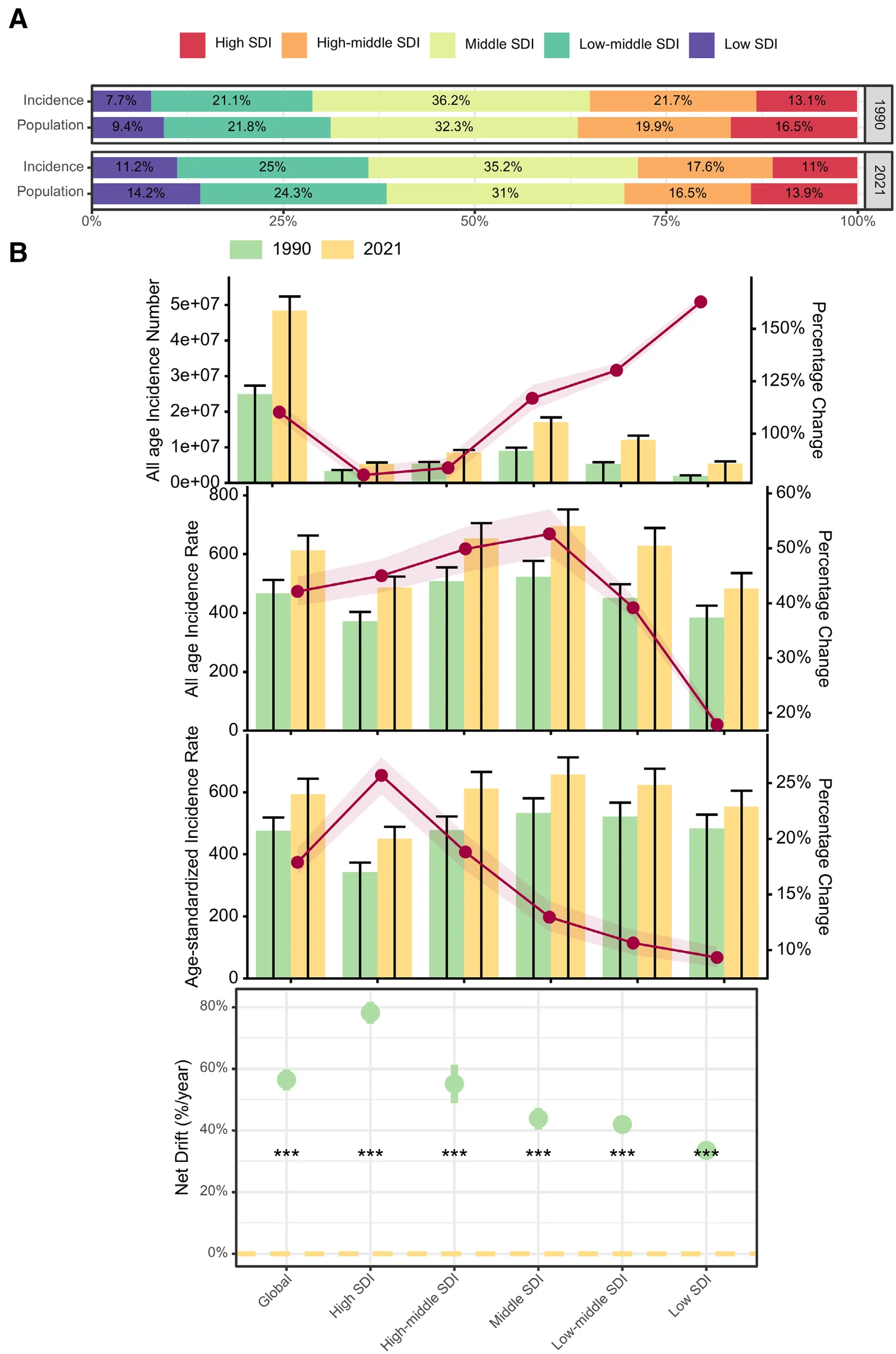
Global rise in NAFLD incidence rates. (A) Variations in the share of NAFLD-related deaths across 5 SDI regions compared to the population proportion of each region from 1990 to 2021. (B) NAFLD incidence rate trends by SDI quintiles from 1990 to 2021.APC = age-period-cohort, NAFLD = nonalcoholic fatty liver disease, SDI = socio-demographic index.

**Table 1:** Trends in nonalcoholic fatty liver disease across SDI quintiles, 1990 to 2021.

Location	Population		All-age numbers			All-age incidence			Age-standardized incidence			NetDrift of incidence from APC model, %/ yr
	Number in 1990	Number in 2021	Number in 1990	Number in 2021	Percent change 1990–2021, %	Rate in 1990	Rate in 2021	Percent change 1990–2021, %	Rate in 1990	Rate in 2021	Percent change 1990–2021, %	
Global	5,333,623,425.64 (5,231,035,879.11–5,444,650,282.47)	7,891,353,300.74 (7,666,733,980.42–8,131,224,517.81)	24,856,158.66 (22,579,697.49–27,333,109.87)	48,353,272.35 (44,229,138.51–52,358,017.44)	1.1 (1.06–1.14)	466.03 (423.35–512.47)	612.74 (560.48–663.49)	0.42 (0.4–0.45)	475.54 (432.59–518.19)	593.28 (542.72–643.7)	0.18 (0.17–0.19)	0.56 (0.53–0.6)
Low SDI	501,300,581.51 (487,907,019.65–514,756,128.18)	1,117,382,590.51 (1,068,353,861.44–1,166,401,098.73)	1,925,958.28 (1,735,020.47–2,127,906.34)	5,396,909.32 (4,857,493.86–5,987,672.28)	1.63 (1.6–1.65)	384.19 (346.1–424.48)	483 (434.72–535.87)	0.18 (0.17–0.19)	483.42 (440.22–527.63)	553.74 (503.53–605.08)	0.09 (0.08–0.1)	0.33 (0.32–0.35)
Low-middle SDI	1,161,410,303.56 (1,120,137,771.59–1,200,684,355.12)	1,921,112,323.91 (1,821,413,930.18–2,023,272,944.93)	5,235,751.86 (4,734,019.63–5,777,133.43)	12,064,461.85 (10,980,813.99–13,233,265.32)	1.3 (1.27–1.34)	450.81 (407.61–497.42)	627.99 (571.59–688.83)	0.39 (0.37–0.41)	520.74 (474.21–566.64)	623.22 (569.61–676.22)	0.11 (0.1–0.12)	0.42 (0.39–0.44)
High SDI	879,533,669.96 (857,730,319.33–901,199,784.96)	1,094,047,735.61 (1,063,559,815.95–1,125,004,948.37)	3,262,720.6 (2,977,823.55–3,550,597.02)	5,326,140.81 (4,910,092.39–5,729,423.93)	0.8 (0.77–0.84)	370.96 (338.57–403.69)	486.83 (448.8–523.69)	0.45 (0.42–0.48)	342.72 (312.23–373.46)	450.03 (412.2–488.43)	0.26 (0.24–0.27)	0.78 (0.75–0.82)
High-middle SDI	1,063,532,174.05 (1,028,133,499.19–1,100,541,186.46)	1,304,031,282.73 (1,251,357,342.44–1,359,983,272.45)	5,404,817.63 (4,893,944.23–5,906,253.77)	8,509,328.08 (7,857,781.78–9,203,998.94)	0.84 (0.79–0.89)	508.2 (460.16–555.34)	652.54 (602.58–705.81)	0.5 (0.46–0.54)	478.36 (435.28–522.1)	611.29 (557.97–665.56)	0.19 (0.17–0.2)	0.55 (0.49–0.61)
Middle SDI	1,722,906,891.05 (1,667,961,018.75–1,774,137,319.58)	2,448,543,920.68 (2,353,584,070.33–2,542,109,614.73)	9,002,611.37 (8,141,914.15–9,945,635.92)	17,017,312.52 (15,552,700.12–18,416,002.69)	1.17 (1.11–1.23)	522.52 (472.57–577.26)	695 (635.18–752.12)	0.53 (0.49–0.57)	533.69 (485.35–580.65)	656.97 (602.08–712.86)	0.13 (0.12–0.14)	0.44 (0.4–0.47)

Age-standardized deaths rate is computed by direct standardization with global standard population in GBD 2021. Net drifts are estimates derived from the age-period-cohort model and denotes overall annual percentage change in deaths, which captures the contribution of the effects from calendar time and successive birth cohorts. Parentheses for all GBD health estimate indicate 95% uncertainty intervals; parentheses for net drift indicate 95% confidence intervals.

APC = age-period-cohort, GBD = the global burden of disease study, SDI = socio-demographic index.

At the regional level, areas with a middle SDI exhibited the highest AAIR for NAFLD in 2021, with a rate of 695 (95% CI: 635.18–752.12) per 100,000 people, while regions with a low SDI had a lower AAIR of 483 (95% CI: 434.72–535.87) per 100,000 individuals. Moreover, the highest ASIR was recorded in regions with a middle SDI, at 656.97/100,000 (95% CI: 602.08–712.86), whereas the lowest ASIR of 450.03/100,000 (95% CI: 412.2–488.43) was observed in high SDI regions. The APC model’s net drift projections revealed contrasting trends in these regions.

At the national level, China recorded the highest number of NAFLD incidence cases in 2021, totaling 9,572,435.36 (95% CI: 8,788,951.07–10,377,517.46), followed by India (8,267,947.86, 95% CI: 7,485,362.61–9,068,568.69), Indonesia (2,080,647, 95% CI: 1,898,615.39–2,275,132.87), Brazil (1,545,088.94, 95% CI: 1,422,183.53–1,665,108.04), and the USA (1,502,212.17, 95% CI: 1,374,518.06–1,626,330.09). Qatar had the highest AAIR of 1309.14 (95% CI: 1163.31–1470.03) per 100,000 individuals, while Denmark reported the lowest AAIR at 341.52 (95% CI: 312.86–369.42) per 100,000. In 2021, Egypt had the highest ASIR at 1188.57/100,000 population (95% CI: 1107.18–1268.67), whereas Finland had the lowest ASIR of 310.17 (95% CI: 284.58–338.28) per 100,000. Net drift values showed variation, with Sudan having the highest value of 0.75 (95% CI: 0.69–0.8) and Georgia the lowest at 0.12 (95% CI: 0.04–0.2) (Fig. 2, Table S1, Supplemental Digital Content 1 and Fig. S1, Supplemental Digital Content 2).

### 3.2. Trends in NAFLD incidence across different age groups

A global increase in NAFLD incidence across all age groups from 1990 to 2021 is shown in Figure 3A. The highest annual increase of 0.886% (95% CI: 0.855%–0.917%) was seen in the 25 to 29 age group. After the 45 to 49 age group, the increase in incidence slows progressively, reaching the smallest rise of 0.107% (95% CI: −0.394% to 0.612%) in the 90 to 94 age group. This trend was consistent across high and middle SDI regions, where the most significant rise occurred in the 30 to 34 age group in high SDI countries, showing an annual increase of 1.415% (95% CI: 1.363%–1.467%). The 15 to 19 age group exhibited the largest increase in high-middle, low-middle, and low SDI regions, followed by a gradual decline in subsequent age groups. Before the age of 45, men had higher incidence rates than women; however, this pattern reversed after 45. In high SDI countries, men experienced a smaller increase in incidence rates compared to women, and no significant gender differences were observed in low-middle and low SDI populations.

Figure 3B depicts changes in the age distribution of NAFLD cases over time. In 1990, individuals under 30 accounted for half of global NAFLD cases, with the 20 to 24 age group having the highest proportion. By 2021, there was a marked increase in NAFLD incidence in the 50 to 69 age group, which became the most prevalent group globally. This pattern was observed consistently in high, high-middle, and middle SDI regions, where the most significant increase occurred in the 50 to 69 age group. In contrast, low and low-middle SDI regions showed little change in age-specific trends. Figure S2, Supplemental Digital Content 3 offers a comprehensive overview of age-specific NAFLD incidence patterns across different countries.

### 3.3. Impact of the APC model on NAFLD incidence rates

An analysis of the APC effects on NAFLD incidence, grouped by SDI quintiles, is presented in Figure 4. Age-related trends were largely similar across all SDI quintiles, with the exception of high SDI regions. In global populations, NAFLD incidence rates exhibited 2 age distribution peaks. The 1st peak occurred in the 20 to 24 age group globally, while in high SDI regions, it shifted to the 15 to 19 age group, which had the highest incidence rate overall. A 2nd peak was observed in the 65 to 69 age group, followed by a decline with increasing age, and a slight rise in the 90 to 94 age group (Fig. 4A). Gender differences were also evident globally, with men under 50 having significantly higher incidence rates than women, while the trend reversed after 50, with women exhibiting higher rates, particularly in different SDI regions.

The period effect globally indicated a continuous increase in the risk of NAFLD over time (Fig. 4B), a pattern consistent across all regions. Minimal gender differences were observed in period effects, except for high-middle and middle SDI regions, where men exhibited higher incidence rates than women, especially before 2003, underscoring regional variations.

Globally, the cohort effect showed a steady increase in NAFLD risk among more recent birth cohorts, with the highest risk observed in the 2000 cohort. Comparable trends were observed across high, high-middle, low-middle, and low SDI regions. In the middle SDI region, birth cohorts born before 1950 exhibited relatively stable risks, but a marked increase in risk was seen in those born after 1950, aligning with younger age groups (Fig. 4C). For cohorts born before 1950, men had significantly higher incidence risks than women, but this gender difference diminished in later cohorts, except in middle SDI regions where men continued to have higher risks. Figures S3, S4 and S5, Supplemental Digital Content 4 provide additional details on the APC effects on NAFLD incidence across different countries.

## 4. Discussion

NAFLD has emerged as a major public health concern globally, primarily due to its association with metabolic disorders such as obesity and type 2 diabetes. Unlike many other liver diseases, NAFLD is not directly linked to alcohol consumption, making its management and prevention heavily reliant on lifestyle modifications and public health interventions. The disease’s potential to progress to more severe forms, such as nonalcoholic steatohepatitis and cirrhosis, underscores the need for early detection and intervention, as highlighted in an analysis.^[16]^ While prevalence studies provide a snapshot of the disease burden at a particular time, incidence data offer insights into the rate at which new cases are occurring. This is crucial for understanding the dynamics of the disease, identifying emerging trends, and evaluating the effectiveness of preventive measures, as noted in a systematic review.^[17]^ The lack of comprehensive incidence data for NAFLD has hindered a thorough understanding of its epidemiology and the development of targeted public health strategies.

From 1990 to 2021, the global incidence of NAFLD has more than doubled, from approximately 24.9 million cases (95% CI: 22.6–27.3 million) to 48.4 million cases (95% CI: 44.2–52.4 million), as reported in our study using data from the GBD 2021 database. ASIR has also increased, with an ASIR of 593.28/100,000 individuals (95% CI: 542.72–643.7) in 2021, indicating a rising risk even after accounting for population aging. Middle SDI regions, such as parts of Asia and Latin America, bear the highest disease burden, with both AAIR and ASIR being higher compared to high SDI regions like North America and Europe. For example, China recorded the highest number of NAFLD incidence cases in 2021, totaling 9.6 million (95% CI: 8.8–10.4 million), followed by India with 8.3 million (95% CI: 7.5–9.1 million), as per our findings. At the national level, Qatar had the highest AAIR of 1309.14/100,000 (95% CI: 1163.31–1470.03), while Egypt had the highest ASIR at 1188.57/100,000 (95% CI: 1107.18–1268.67), highlighting regional variations.

Age-specific trends show that the highest increase in NAFLD incidence globally occurred in the 25 to 29 age group, with an annual increase of 0.886% (95% CI: 0.855%–0.917%), as depicted in our results. Over time, there was a marked shift in age distribution, with the 50 to 69 age group becoming the most prevalent by 2021, up from younger age groups like 20 to 24 in 1990.

Several factors may contribute to the observed trends in NAFLD incidence. Among these, the global increase in obesity and other metabolic abnormalities is likely an important contributor. Obesity is a well-established risk factor for NAFLD, and a meta-analysis of 21 cohort studies including 381,655 participants found that obesity was associated with a 3.53-fold higher risk of developing NAFLD, with a dose-dependent increase in risk with increasing BMI.^[18]^ Consistent with this association, a global systematic review and meta-analysis reported a high prevalence of NAFLD among overweight and obese populations, with pooled estimates of 69.99% and 75.27%, respectively.^[19]^ However, the relationship between obesity and NAFLD is unlikely to be uniform across countries and population subgroups. Global analyses have demonstrated substantial increases in overweight and obesity over recent decades, accompanied by considerable heterogeneity across countries and population groups.^[9]^ Such differences in the magnitude and timing of the obesity epidemic may partly contribute to the geographic and temporal heterogeneity in NAFLD incidence observed in our study. In particular, rapid urbanization, dietary transition, and changes in physical activity in many middle-income settings may have increased exposure to obesogenic environments, potentially contributing to the relatively high NAFLD incidence observed in middle SDI regions. Socioeconomic circumstances may further influence population-level exposure to obesity-related risk factors, as lower neighborhood socioeconomic status has been associated with higher odds of overweight and obesity in a systematic review and meta-analysis of >1.2 million individuals.^[20]^ Nevertheless, these relationships are complex and may also be influenced by differences in healthcare access, diagnostic capacity, dietary patterns, and other metabolic risk factors across settings.

Age-specific patterns may provide additional insight into the observed trends. In our APC analysis, the greatest annual increase in NAFLD incidence was observed among individuals aged 25 to 29 years, while the proportion of incident cases shifted toward older age groups, with the 50 to 69-year group accounting for a greater share in 2021 than in 1990. Evidence from a large prospective cohort study further suggests that weight gain during early adulthood is associated with a substantially increased subsequent risk of NAFLD, supporting the potential relevance of cumulative adiposity exposure across the life course.^[21]^ The increasing risk observed among more recent birth cohorts in our study may therefore partly reflect exposure to an increasingly obesogenic environment across successive generations. However, obesity should not be considered a sufficient explanation for the observed patterns because NAFLD also occurs in individuals who are not obese and may be influenced by metabolic dysfunction, dietary factors, physical inactivity, genetic susceptibility, and other environmental determinants. Therefore, the observed geographic, age-specific, and cohort-specific patterns are likely to reflect the combined effects of obesity and other interacting metabolic and environmental factors rather than obesity alone.

Based on the APC model, the positive net drift of 0.56%/yr (95% CI: 0.53%–0.60%) indicates an overall increase in NAFLD incidence over the study period. The cohort effect also showed higher relative risks among more recent birth cohorts, particularly those born after 1950. These findings highlight the importance of continued surveillance of NAFLD incidence and its metabolic risk factors as these cohorts age.

To address the growing burden of NAFLD, a multifaceted approach is needed, as advocated in a global consensus statement.^[22]^ Public awareness campaigns promoting healthy lifestyles, including balanced diets and regular physical activity, are essential. For example, the Mediterranean diet, known for its low saturated fat content, has been shown to reduce NAFLD risk.^[23]^ Implementing appropriate risk assessment and early detection strategies in high-risk populations, particularly those with obesity or diabetes, may facilitate earlier identification and management of NAFLD.^[24]^ Tailored programs to manage obesity, diabetes, and other metabolic risk factors are crucial, with evidence showing that weight loss can improve or reverse features of NAFLD, emphasizing the importance of lifestyle changes.^[23]^ Regulatory measures to promote healthier food environments and encourage physical activity, such as a low glycemic index Mediterranean diet combined with aerobic exercise, can reduce NAFLD incidence.^[25]^ Continued monitoring of NAFLD incidence and its risk factors is important for evaluating the effectiveness of interventions and identifying emerging trends.

While our study provides valuable insights, several limitations should be acknowledged. First, variations in data availability, case ascertainment, and diagnostic practices across countries may affect the comparability of GBD estimates, and underdiagnosis may be more substantial in low SDI regions because of limited healthcare access and diagnostic capacity. Second, although obesity and other metabolic risk factors are important contributors to NAFLD, the present analysis did not incorporate obesity prevalence or other metabolic exposures as time-varying explanatory variables. Therefore, their independent contributions to the observed temporal, age-specific, and cohort-specific patterns could not be directly quantified. Accordingly, the relationships discussed here should be interpreted as contextual explanations rather than causal associations. Future longitudinal studies integrating harmonized data on obesity, metabolic risk factors, lifestyle characteristics, and NAFLD incidence across countries may help clarify the mechanisms underlying the observed heterogeneity in NAFLD incidence trends.

## 5. Conclusion

In conclusion, this study highlights the increasing global burden of NAFLD, particularly in middle-income countries, and identifies key age, period, and cohort effects that have contributed to this trend. The results highlight the importance of implementing focused public health strategies to tackle the modifiable risk factors linked to NAFLD and lower its incidence. Future studies should further investigate the root causes of NAFLD and assess the success of preventive and therapeutic approaches in reducing the impact of this increasing public health concern.

**Figure 2.**
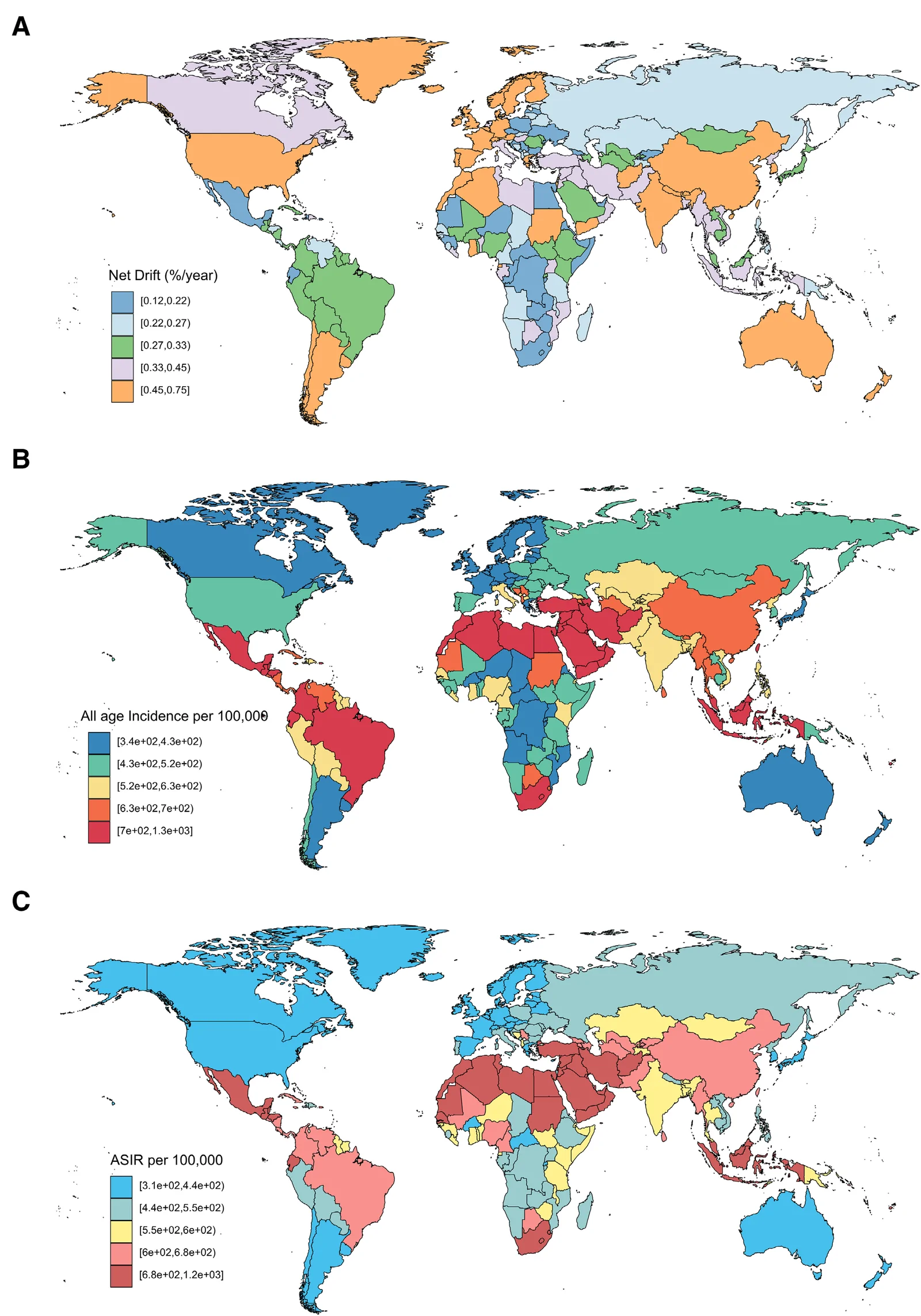
Net drift (A), AAIR (B), and ASIR (C) across 204 countries and territories. (A) Global map showing the net drifts in NAFLD incidence rates, reflecting the trends linked to calendar years and successive birth cohorts. The global net drift for NAFLD incidence rate was 0.564% (95% CI: 0.530–0.598). (B) World map displaying the AAIR for NAFLD in 2021, with a global AAIR of 612.74 (95% CI: 560.48–663.49) per 100,000 individuals. (C) World map of ASIR for NAFLD in 2021, with a global ASIR of 593.28 (95% CI: 542.72–643.7) per 100,000 individuals.AAIR = all-age incidence rate, ASIR = age-standardized incidence rate, CI = confidence interval, NAFLD = nonalcoholic fatty liver disease.

**Figure 3.**
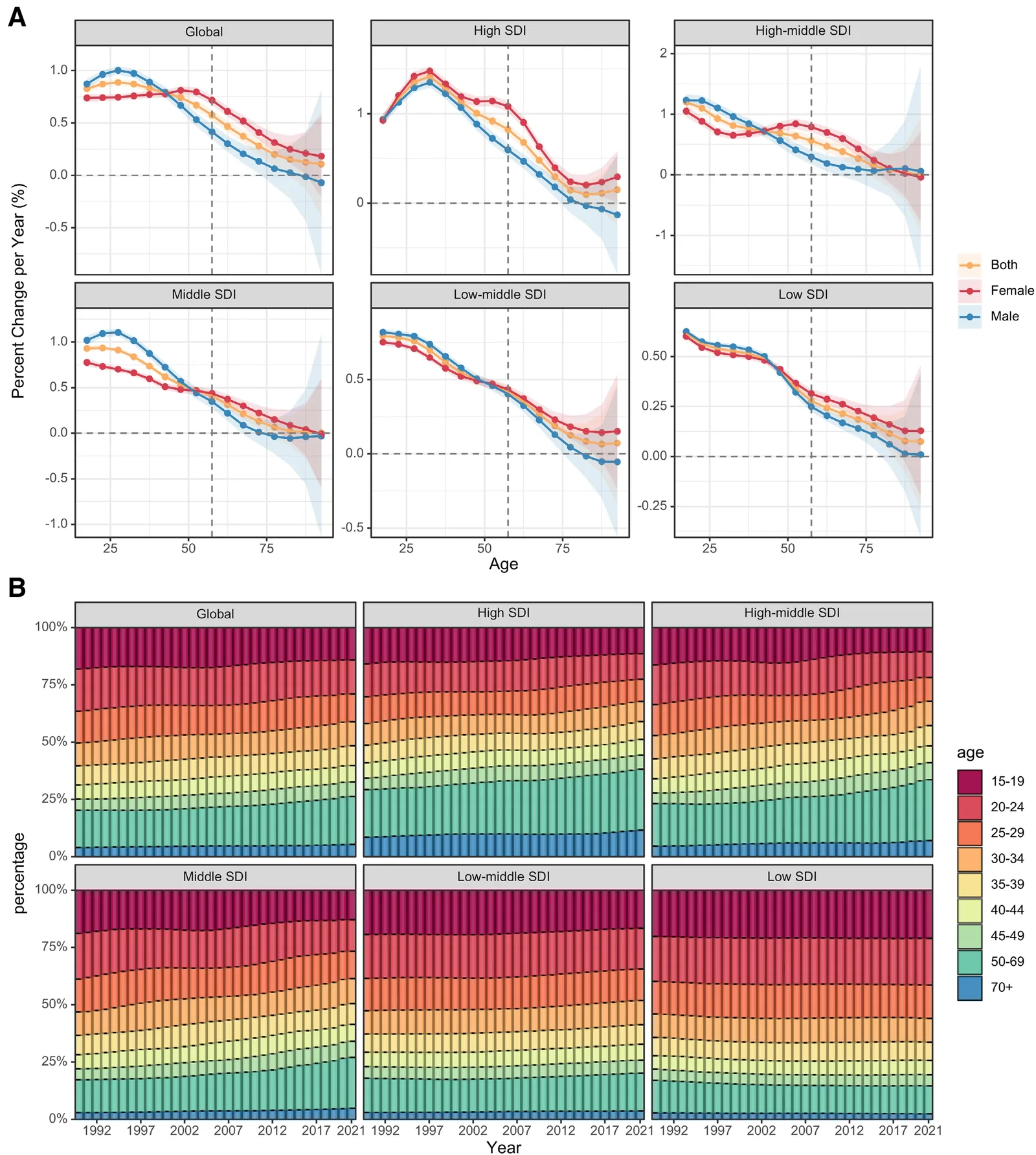
Local drifts of incidence rates and age distribution of NAFLD cases by SDI quintiles from 1990 to 2021. (A) Local drifts in NAFLD incidence rates (estimated from age-period-cohort models) across 16 age groups (15–19, 20–24, 25–29, …, 90–94 years) from 1990 to 2021. The points and shaded regions represent the annual percentage change in incidence rate (%/yr) with the corresponding 95% confidence interval (CI). (B) Changes over time in the relative distribution of NAFLD incidences across various age groups (15–19, 20–24, 25–29, 30–34, 35–39, 40–44, 45–49, 50–69, 70+ years) from 1990 to 2021. ASIR = age-standardized incidence rate, CI = confidence interval, NAFLD = nonalcoholic fatty liver disease, SDI = socio-demographic index.

**Figure 4.**
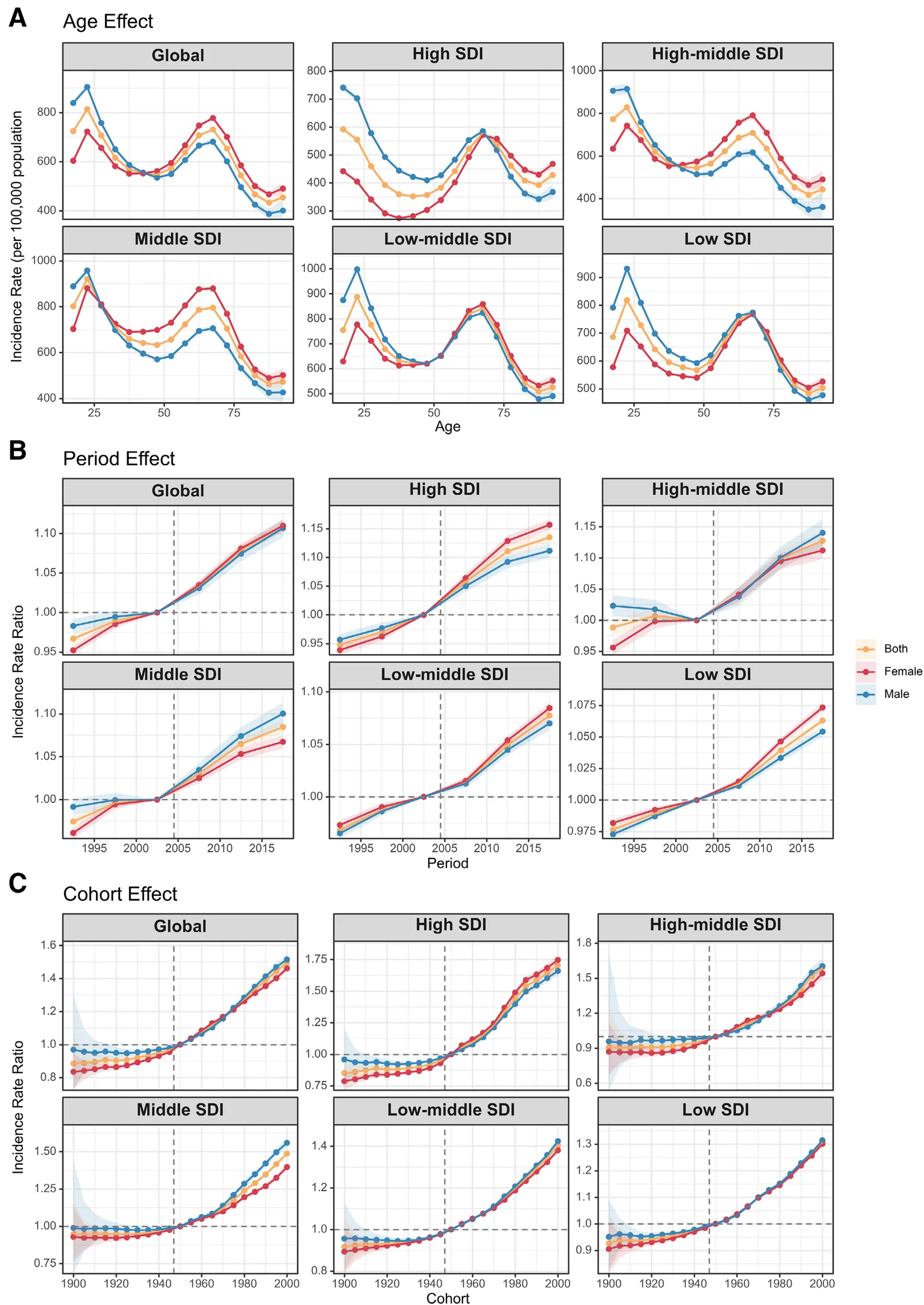
The effects of age, period, and cohort on NAFLD incidence across SDI quintiles. (A) Age effects represented by the fitted longitudinal age curves of incidence (per 100,000 person-years), adjusted for period variations. (B) Period effects indicated by the relative risk of incidence (incidence rate ratio), calculated as the ratio of age-specific rates from the 1990 to 1994 period (with 2000–2004 as the reference period) to 2015 to 2019. (C) Cohort effects presented by the relative risk of incidence, calculated as the ratio of age-specific rates between the 1900 and 2000 cohorts, with the 1950 cohort as the reference. The points and shaded areas indicate incidence rates or rate ratios along with their corresponding 95% confidence intervals (CI). CI = confidence interval, NAFLD = nonalcoholic fatty liver disease, SDI = socio-demographic index.

## Acknowledgements

We would like to express thanks to the Global Burden of Disease study for making this research possible through the provision of publicly available data.

## Author contributions

**Conceptualization:** Meiying Zheng, Luyao Gong, Zhaojie Su.

**Data curation:** Meiying Zheng, Zhaojie Su.

**Formal analysis:** Haiying Chen, Luyao Gong.

**Investigation:** Meiying Zheng, Meng Zou, Zhaojie Su.

**Methodology:** Meiying Zheng, Meng Zou, Haiying Chen, Na Wang.

**Project administration:** Meiying Zheng, Meng Zou, Luyao Gong, Zhaojie Su.

**Resources:** Meng Zou, Haiying Chen, Na Wang, Zhaojie Su.

**Software:** Meiying Zheng, Haiying Chen, Zhaojie Su.

**Supervision:** Meiying Zheng, Zhaojie Su.

**Validation:** Meiying Zheng, Luyao Gong, Na Wang.

**Visualization:** Na Wang.

**Writing – original draft:** Meiying Zheng, Meng Zou, Haiying Chen, Luyao Gong, Na Wang, Zhaojie Su.

**Writing – review & editing:** Meiying Zheng, Meng Zou, Haiying Chen, Luyao Gong, Na Wang, Zhaojie Su.












