# Supplementary material for: Three-decade trends in nonalcoholic fatty liver disease across 204 countries and territories: An age-period-cohort analysis from 1990 to 2021
Source: Medicine (Baltimore). 2026 Oct 2;105(40):e50989. doi: 10.1097/MD.0000000000050989 (PMC13632965; doi:10.1097/MD.0000000000050989)
Supplement: Supplementary file 2 [file medi-105-e50989-s003.pdf]

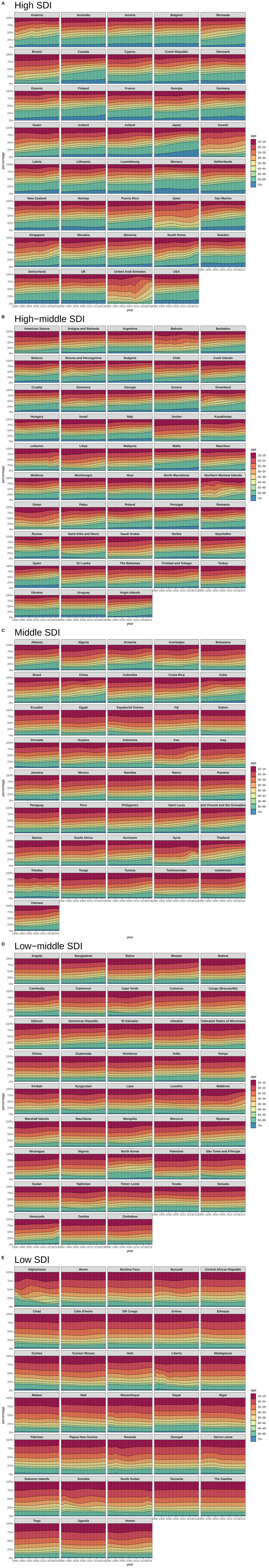

A
High SDI
Andorra
Australia
Austria
Belgium
Bermuda
Brunei
Canada
Cyprus
Czech Republic
Denmark
Estonia
Finland
France
Georgia
Germany
Guam
Iceland
Ireland
Japan
Kuwait
Latvia
Lithuania
Luxembourg
Monaco
Netherlands
New Zealand
Norway
Puerto Rico
Qatar
San Marino
Singapore
Slovakia
Slovenia
South Korea
Sweden
Switzerland
UK
United Arab Emirates
USA
B
High−middle SDI
American Samoa
Antigua and Barbuda
Argentina
Bahrain
Barbados
Belarus
Bosnia and Herzegovina
Bulgaria
Chile
Cook Islands
Croatia
Dominica
Georgia
Greece
Greenland
Hungary
Israel
Italy
Jordan
Kazakhstan
Lebanon
Libya
Malaysia
Malta
Mauritius
Moldova
Montenegro
Niue
North Macedonia
Northern Mariana Islands
Oman
Palau
Poland
Portugal
Romania
Russia
Saint Kitts and Nevis
Saudi Arabia
Serbia
Seychelles
Spain
Sri Lanka
The Bahamas
Trinidad and Tobago
Turkey
Ukraine
Uruguay
Virgin Islands
C
Middle SDI
Albania
Algeria
Armenia
Azerbaijan
Botswana
Brazil
China
Colombia
Costa Rica
Cuba
Ecuador
Egypt
Equatorial Guinea
Fiji
Gabon
Grenada
Guyana
Indonesia
Iran
Iraq
Jamaica
Mexico
Namibia
Nauru
Panama
Paraguay
Peru
Philippines
Saint Lucia
Saint Vincent and the Grenadines
Samoa
South Africa
Suriname
Syria
Thailand
Tokelau
Tonga
Tunisia
Turkmenistan
Uzbekistan
Vietnam
D
Low−middle SDI
Angola
Bangladesh
Belize
Bhutan
Bolivia
Cambodia
Cameroon
Cape Verde
Comoros
Congo (Brazzaville)
Djibouti
Dominican Republic
El Salvador
eSwatini
Federated States of Micronesia
Ghana
Guatemala
Honduras
India
Kenya
Kiribati
Kyrgyzstan
Laos
Lesotho
Maldives
Marshall Islands
Mauritania
Mongolia
Morocco
Myanmar
Nicaragua
Nigeria
North Korea
Palestine
São Tomé and Príncipe
Sudan
Tajikistan
Timor−Leste
Tuvalu
Vanuatu
Venezuela
Zambia
Zimbabwe
E
Low SDI
Afghanistan
Benin
Burkina Faso
Burundi
Central African Republic
Chad
Côte d'Ivoire
DR Congo
Eritrea
Ethiopia
Guinea
Guinea−Bissau
Haiti
Liberia
Madagascar
Malawi
Mali
Mozambique
Nepal
Niger
Pakistan
Papua New Guinea
Rwanda
Senegal
Sierra Leone
Solomon Islands
Somalia
South Sudan
Tanzania
The Gambia
Togo
Uganda
Yemen
percentage
100%
75%
50%
25%
0%
year
1990 1995 2000 2005 2010 2015 2019
age
15−19
20−24
25−29
30−34
35−39
40−44
45−49
50−69
70+
