# Supplementary material for: Three-decade trends in nonalcoholic fatty liver disease across 204 countries and territories: An age-period-cohort analysis from 1990 to 2021
Source: Medicine (Baltimore). 2026 Oct 2;105(40):e50989. doi: 10.1097/MD.0000000000050989 (PMC13632965; doi:10.1097/MD.0000000000050989)

A High SDI

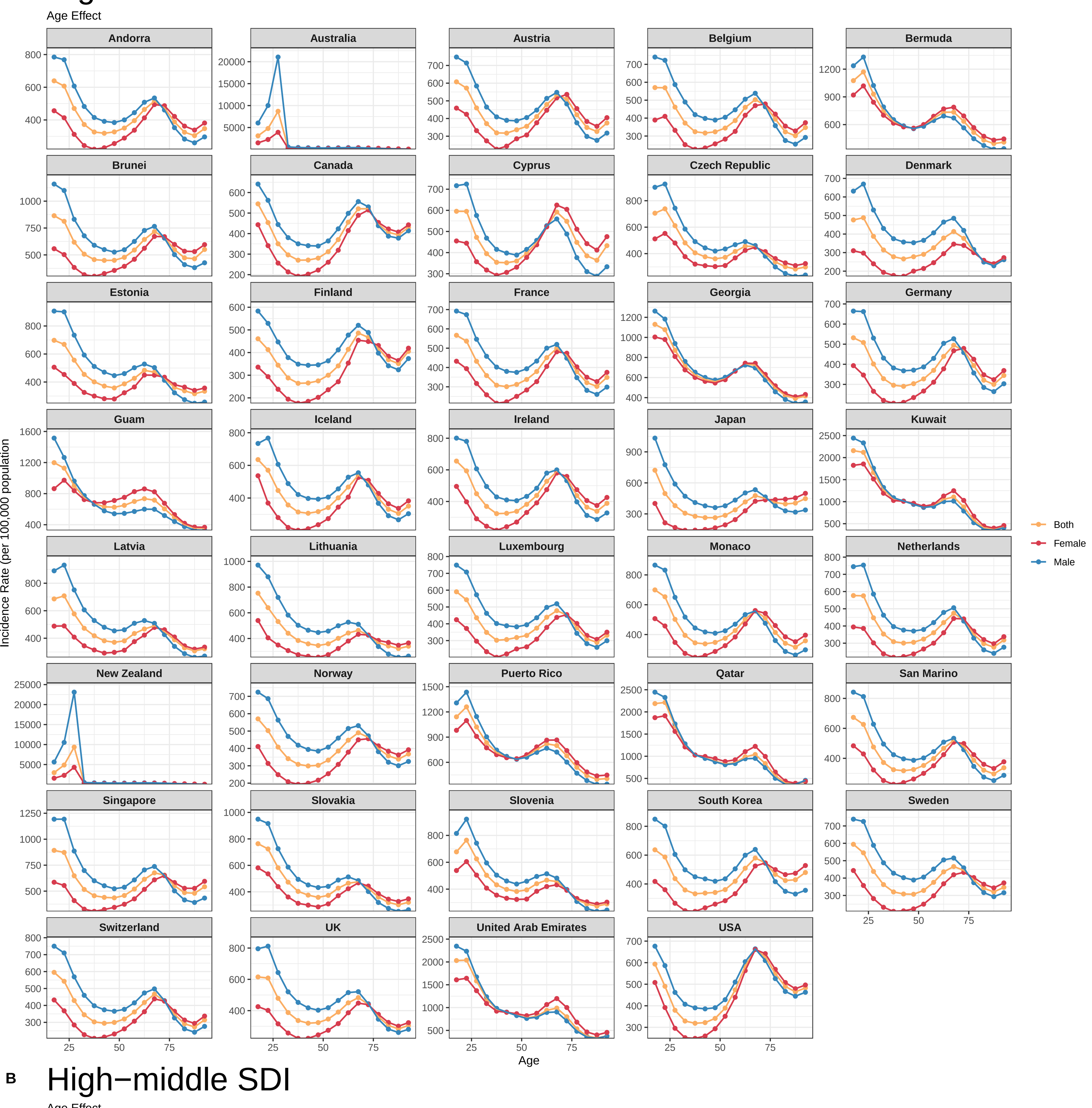

B High−middle SDI

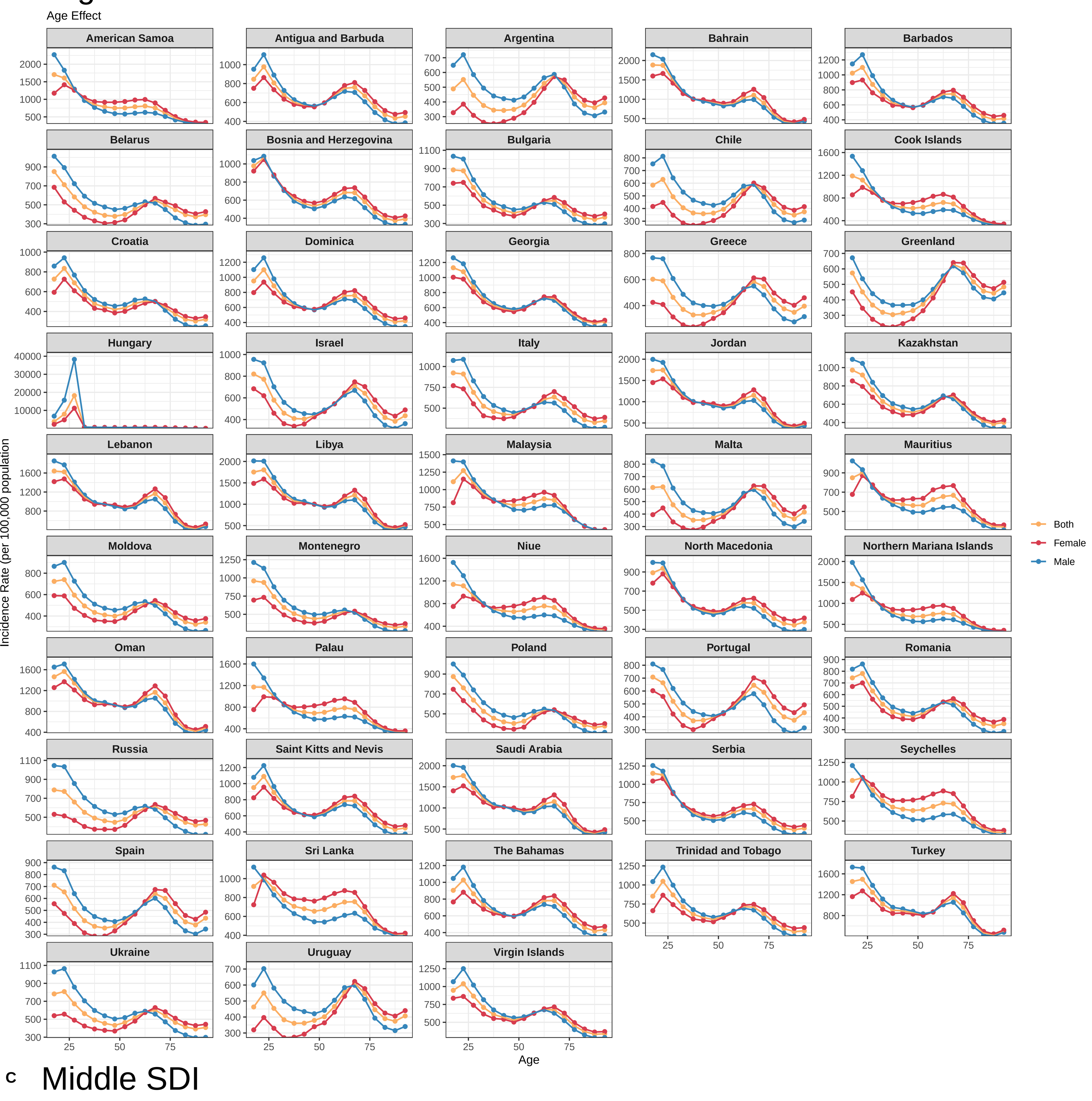

C Middle SDI

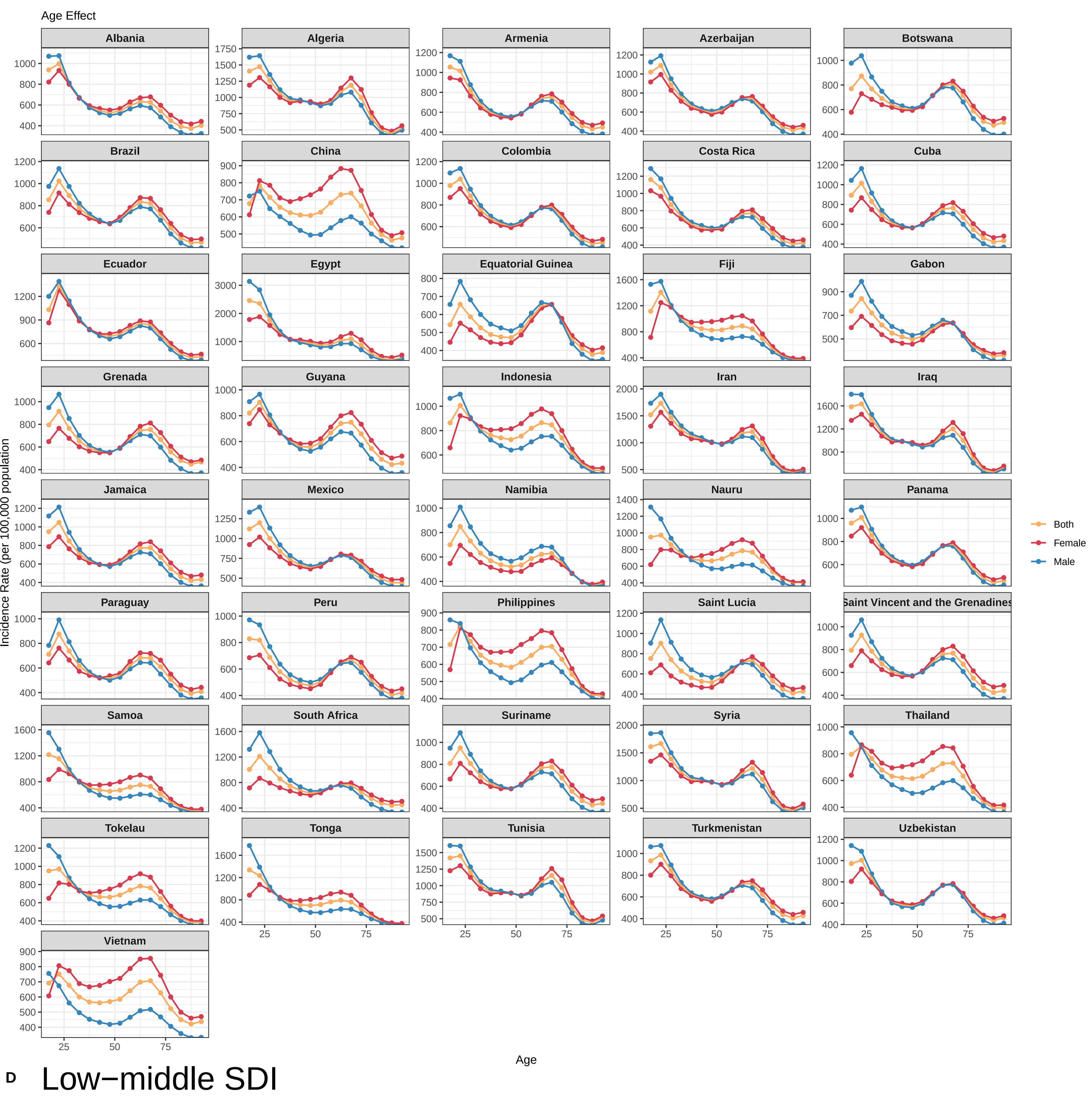

D Low−middle SDI

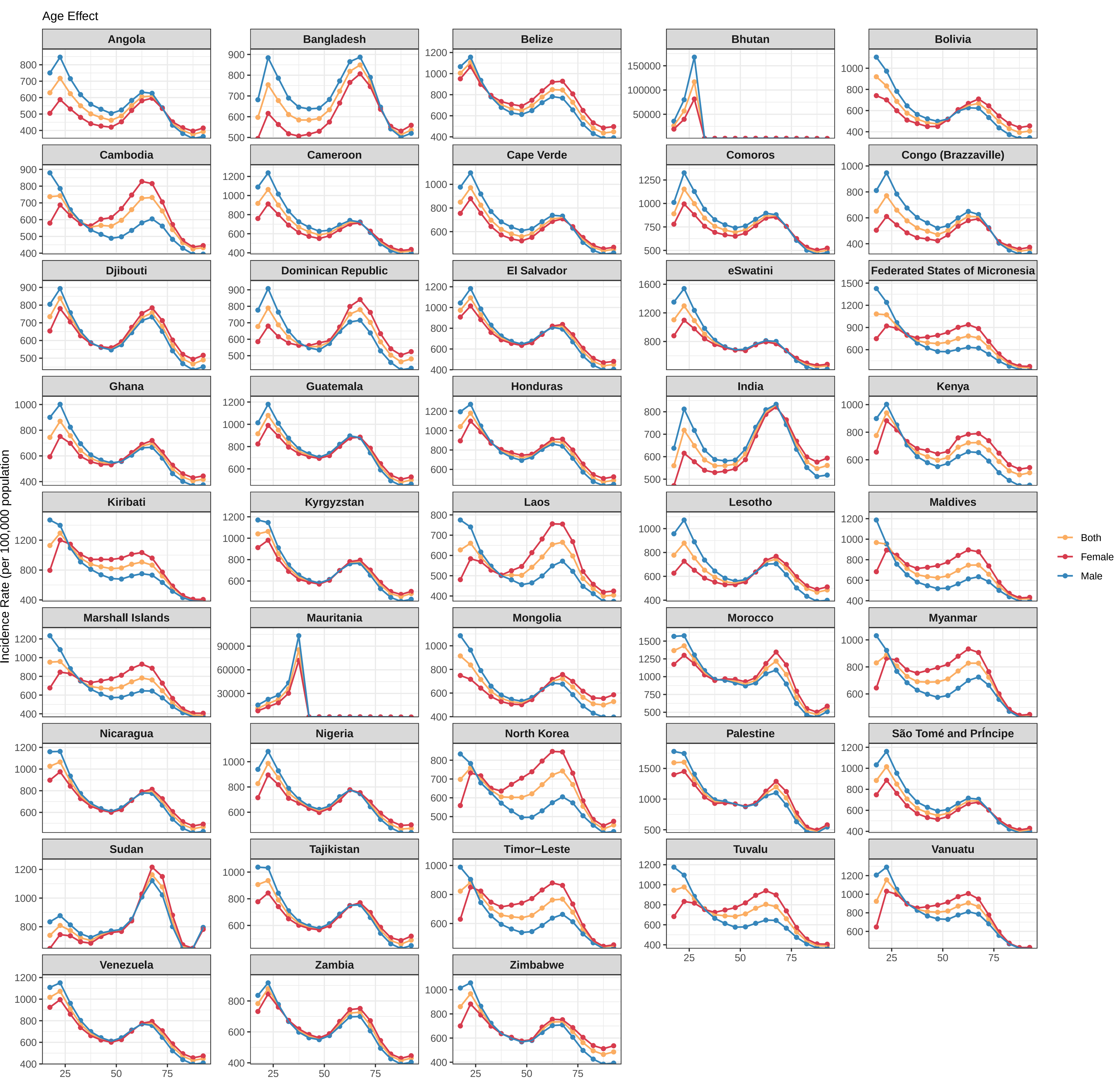

E Low SDI

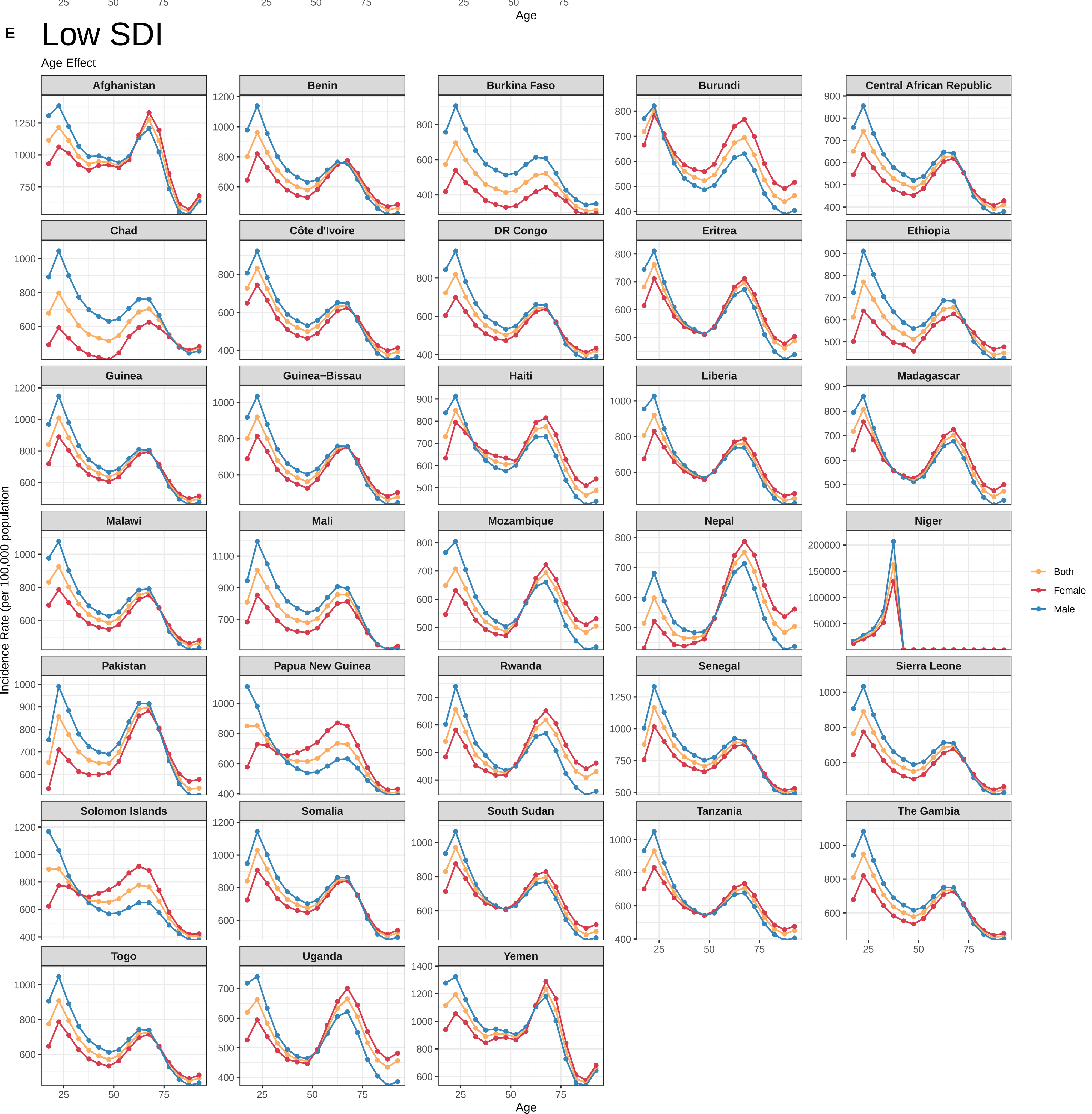

Supplement: Supplementary file 3 [file medi-105-e50989-s004.pdf]
